# *Methylobacterium populi* VP2: Plant Growth-Promoting Bacterium Isolated from a Highly Polluted Environment for Polycyclic Aromatic Hydrocarbon (PAH) Biodegradation

**DOI:** 10.1155/2014/931793

**Published:** 2014-08-03

**Authors:** Valeria Ventorino, Filomena Sannino, Alessandro Piccolo, Valeria Cafaro, Rita Carotenuto, Olimpia Pepe

**Affiliations:** ^1^DIA-Dipartimento di Agraria, Università degli Studi di Napoli Federico II, Via Università 100, 80055 Portici, Italy; ^2^Dipartimento di Biologia, Università degli Studi di Napoli Federico II, Complesso Universitario Monte S. Angelo, Via Cintia 4, 80126 Napoli, Italy

## Abstract

The use of microorganisms to accelerate the natural detoxification processes of toxic substances in the soil represents an alternative ecofriendly and low-cost method of environmental remediation compared to harmful incineration and chemical treatments. Fourteen strains able to grow on minimal selective medium with a complex mixture of different classes of xenobiotic compounds as the sole carbon source were isolated from the soil of the ex-industrial site ACNA (Aziende Chimiche Nazionali Associate) in Cengio (Savona, Italy). The best putative degrading isolate, *Methylobacterium populi* VP2, was identified using a polyphasic approach on the basis of its phenotypic, biochemical, and molecular characterisation. Moreover, this strain also showed multiple plant growth promotion activities: it was able to produce indole-3-acetic acid (IAA) and siderophores, solubilise phosphate, and produce a biofilm in the presence of phenanthrene and alleviate phenanthrene stress in tomato seeds. This is the first report on the simultaneous occurrence of the PAH-degrading ability by *Methylobacterium populi* and its multiple plant growth-promoting activities. Therefore, the selected indigenous strain, which is naturally present in highly contaminated soils, is good candidate for plant growth promotion and is capable of biodegrading xenobiotic organic compounds to remediate contaminated soil alone and/or soil associated with plants.

## 1. Introduction

The accumulation of large amounts of persistent chemicals in the soil from urban, industrial, and agricultural activities is an increasingly important worldwide problem. In Italy, the ex-industrial site of ACNA (Aziende Chimiche Nazionali Associate) in Cengio (Savona, Italy) was contaminated by different classes of organic compounds and is on the list of national priorities for environmental reclamation.

Among the most common pollutants in contaminated environments, polycyclic aromatic hydrocarbons (PAHs) are fused-ring aromatic compounds that exist in nature as a result of anthropogenic polluting events such as waste combustion, industrial treatment, and petroleum processing [1]. PAHs have been recognised to be mutagenic, carcinogenic, and cytotoxic to humans and animals [2]; therefore, their presence in contaminated soils represents a significant risk to the environment due to their intrinsic chemical stability, lower solubility, and high recalcitrance to different types of degradation [3]. For this reason, the United States Environmental Protection Agency (US EPA) considers PAHs to be priority pollutants for remediation.

Numerous physical and chemical technologies, such as soil washing, have been developed to remove PAHs from the environment and to remediate contaminated soils. However, these methods are expensive and only partially effective [4]. In this context, bioremediation appears to be a valid, environmentally friendly, and cost-competitive approach for the clean-up of PAH-polluted sites and to achieve permanent detoxification of those sites. This appealing technology is based on natural degradation carried out by* in situ* stimulation of indigenous microorganisms present in the polluted soils [5, 6] and/or by the inoculation of selected microorganisms that are able to remove the contaminants by mineralising the PAHs and/or forming metabolites that are less toxic than the original pollutants [7]. In fact, bacteria have evolved several mechanisms to overcome the very low water solubility of PAHs, such as bioemulsifier production, membrane carriers, and the ability to grow in biofilms on hydrophobic surfaces [8]. In particular, biofilms are assemblages of surface-associated microbial cells that are enclosed in an extracellular polymeric matrix made primarily of a polysaccharide material [9]. They are considered to be the prevailing microbial life form in most environments [10, 11] and allow the bacteria to enjoy a number of advantages, such as increased resistance to antimicrobial agents [12–15]. However, the use of strains isolated from other environments is not always effective because the efficiency of the inoculum depends on several factors, including the site conditions; the indigenous microbial populations; and the type, concentration, bioavailability, and toxicity of the pollutants present in the soil [16]. In addition, when microorganisms are added, the duration of assessment and biological process efficiency depend on the evolution of the bacterial communities in terms of composition and catabolic activity [17].

Recently, the use of plant growth-promoting rhizobacteria (PGPR) associated with plants for environmental remediation has emerged as a promising field, although very few field studies have been performed [18]. PGPR are able to benefit plant development using a wide range of mechanisms, including synthesising compounds to promote plant growth and/or increasing the uptake of nutrients and acting as biocontrol agents by suppressing plant pathogens in the rhizosphere. In addition, plant growth-promoting microorganisms could contribute to the remediation process via multiple modes of action [19] because these microorganisms can both degrade and/or mineralise organic xenobiotic compounds, allowing them to serve directly as contaminant degraders [20] or in combination with plants [18, 21]. In fact, synergistic action of both the rhizosphere microorganisms and the plants can lead to increased availability of hydrophobic compounds, affecting their removal and/or degradation [22]. In addition, during microbe-assisted phytoremediation, the plant growth-promoting microbial strains could increase the tolerance of plants to contaminants, both enhancing plant germination and root biomass accumulation through the biosynthesis of phytohormones [23] and degrading the xenobiotic compounds before they negatively impact the plants [24].

On the basis of these considerations, indigenous plant growth-promoting microbial strains were isolated, characterised, and selected from highly contaminated environments to evaluate their potential use in bioremediation and/or a microbe-assisted phytoremediation project.

## 2. Materials and Methods

### 2.1. Soil Description

The soil sample was collected from the site of ACNA (Aziende Chimiche Nazionali Associate), an industrial area of Cengio (near Savona) in northern Italy. The site was extremely polluted due to the irregular disposal of organic and inorganic contaminants on the surface and lower soil horizons since 1882. The pollution of the area has intensified since the manufacturing of a range of organic colorants that began in 1939. In 1999, due to the serious contamination of the surrounding soils and waters, the ACNA site was included in the list of national priorities for environmental remediation. The chemical and physical properties of the soil were previously reported by Conte et al. [25].

### 2.2. Soil Contaminants

Contaminated soil aqueous extract (CSAE) was obtained as previously reported [26]. Briefly, according to this method, a Soxhlet extraction system under reflux with an acetone/*n*-hexane (50 : 50, v : v) mixture was employed. The organic extracts were dried by rotavapor and dissolved in an acetone/water solution (5 : 95, v : v). Then, solid phase extraction (SPE) was performed. Finally, the elutes were analysed through GC/MS to evaluate the concentration of the contaminants. The soil contaminants identified in the GC/MS chromatograms were previously reported [26].

### 2.3. Microbial Isolation and Enrichment Methods

The soil samples (20 g) were shaken for 30 min in 180 mL of quarter-strength Ringer's solution (Oxoid, Milan, Italy) containing 0.324 g of tetrasodium pyrophosphate to detach the bacteria from the soil particles. After the soil particles were allowed to settle for 15 min, the solution was diluted tenfold in series. Potential PAH-degrading microorganisms were isolated by spreading the serial dilutions on a minimal selective solid medium (MSSM), containing 1% natural nutrients (soil extract obtained from freshly collected meadow soil) [27], 0.5% contaminated soil aqueous extract (CSAE) obtained as above reported, and 1.5% agar bacteriological (Oxoid). The plates were incubated at 28°C for 15 days.

The isolated colonies that were able to grow by streaking on a minimal solid medium containing 1.5% CSAE as the sole carbon source were further selected.

A serial enrichment strategy was carried out that inoculated the selected microbial isolates in a minimal selective liquid medium (MSLM) containing increasing concentrations of CSAE (up to 50%), with or without the addition of nutrients (1% soil extract). The growth of the isolates in the liquid culture was determined by a spread plate count method using MSSM containing the same amount of CSAE (up to 50%).

### 2.4. Identification of the Selected Putative PAH-Degrading Microorganisms

The putative selected PAH-degrading bacterial strains were identified using a polyphasic approach on the basis of their phenotypic, biochemical, and molecular characterisation.

A colony morphology analysis was carried out observing the shape, edge, dimension, elevation, consistency, and colour of the cells. Catalase activity was detected by evaluating the effervescence production after dissolving the colony in 3% hydrogen peroxide. Oxidase activity was detected using oxidase strips (Oxoid). A Gram reaction was performed using the KOH test as described by Halebian et al. [28]. The micromorphology of the cells was observed using an optic microscope (Eclipse E200; Nikon).

A carbon source utilisation test was performed using a standard protocol as described by Green and Bousfield [29] and different carbon sources (fructose, methane, methylamine, ethanol, cyanate, thiocyanate, glucose, and citrate).

Molecular identification was performed by sequencing the 16S rRNA gene. The total genomic DNA was extracted and purified by the InstaGene Matrix (Bio-Rad Laboratories, Milan, Italy) according to the supplier's recommendations. Approximately 50 ng of DNA was used as a template for the PCR assay.

The synthetic oligonucleotide primers fD1 (5′-AGAGTTTGATCCTGGCTCAG-3′) and rD1 (5′-AAGGAGGTGATCCAGCC-3′) were used to amplify the 16S rRNA gene [30]. The PCR mixture was prepared as reported by Alfonzo et al. [31]. The PCR conditions were performed as described by Palomba et al. [32].

The presence of the PCR products was verified by agarose (1.5% wt/vol) gel electrophoresis at 100 V for 1 h, and the products were purified using a QIAquick gel extraction kit (Qiagen S.p.A, Milan, Italy) and sequenced. The DNA sequences were determined and analysed as previously reported [33], and they were compared to the GenBank nucleotide data library using the Blast software at the National Centre of Biotechnology Information website (http://www.ncbi.nlm.nih.gov/Blast.cgi) [34].

The sequence obtained has been deposited in the GenBank database under accession number KF955558.

Multiple nucleotide alignments of the nearly full-length 16S rRNA gene of the bacterial strain and 32 type strains were carried out using the Clustal W program [35] from the MEGA version 4.0 [36]. The nucleotide sequences of the type strains were retrieved from the Ribosomal Database Project (RDP, http://rdp.cme.msu.edu/). The phylogenetic tree was inferred using the neighbor-joining method with a maximum composite likelihood model in the MEGA4 program with bootstrap values based on 1,000 replications.

### 2.5. Biofilm Analysis

The biofilm formation was analysed in M9 medium [37] supplemented with low-viscosity paraffin (LVP) as an organic hydrophobic phase. Phenanthrene (PHE) dissolved in LVP was used as the model PAH compound to test the ability of the strain to respond to the presence of aromatic carbon sources.

The bacterial strain was grown in M9 medium containing 0.5% methanol (M9/MeOH medium) as the carbon source. The cultures were incubated for 72 h at 28°C and then diluted in 10 mL of M9/MeOH medium until they reached an optical density of 0.01 O.D._600 nm_. The cultures were supplemented with 400 *μ*L of low-viscosity paraffin (LVP) or 8 mg of PHE dissolved in 400 *μ*L of low-viscosity paraffin (LVP/PHE) and incubated in 50 mL polypropylene tubes at 28°C under orbital shaking (220 rpm). The control samples were prepared and incubated as described above in the absence of bacteria. After 96 h of incubation, 500 *μ*L of a medium/oil drop emulsions were collected, frozen at −20°C, thawed, and centrifuged at 14,000 ×g for 10 min at 4°C to separate the mixtures into four phases: a cell pellet (planktonic cells), an aqueous phase, a disk of gelatinous (not homogeneous) material, and an oil phase.

### 2.6. Cell Surface Hydrophobicity Test and Emulsification Properties

The ability of the bacterial cells to adhere to the hydrocarbons was used as a measure of their cell surface hydrophobicity (CSH). The assay was performed as described by Mattos-Guaraldi et al. [38] with some modifications. Briefly, the strain was grown in M9/MeOH medium with and without LVP or LVP/PHE as described above. The control samples were prepared and incubated in the absence of the organic phase. The cultures were incubated for 72 h at 28°C under orbital shaking (220 rpm). The water phase (3 mL) of each culture was collected and centrifuged at 5,000 ×g for 20 min at 4°C to separate the cells and the supernatant. The cells were washed twice in 10 mM phosphate buffer, pH 7.0, and suspended in fresh M9 medium at a final optical density at 600 nm of approximately 1 O.D._600 nm_. The cell suspension absorbance at 600 nm was measured (*A*). To test the CSH, 0.1 mL of LVP was added to 1 mL of the cell suspension, and the sample was vortexed vigorously for 2 min. The phases were allowed to separate for 15 min at room temperature. The optical density at 600 nm of the cells remaining in suspension in the water phase (*B*) was then measured. Hydrophobicity (CSH index) was calculated as the proportion of cells that were excluded from the water phase, determined by the following equation: [(*A* − *B*)/*A*] × 100. When the CSH index was ≤20%, the strains were considered hydrophilic; strains with CSH values ranging between 20% and 50% were considered moderately hydrophobic; and highly hydrophobic strains had CSH values ≥50%.

To test the emulsification properties of the free cell medium, the supernatants were further centrifuged at 18,000 ×g for 30 min at 4°C and used without further purification. Typically, 1 mL of the supernatants and 1 mL of LVP were mixed vigorously for 2 min in glass tubes (5 × 100 mm). The emulsions were allowed to stand for 24 h at room temperature. The emulsification index (*E*_24_) was calculated by the following equation: (height of emulsified layer/height of the total oil phase) × 100. An *E*_24_ value of 0% indicated no emulsification whereas an *E*_24_ value of 100% indicated 100% emulsification.

### 2.7. Phenanthrene Degradation

The hydrocarbon degradation experiments were carried out in M9 medium supplemented with PHE crystals. Typically, 10 mL of the M9 medium was supplemented with 2 mg of PHE dissolved in 100 *μ*L of acetone to reach a final concentration of 200 mg L^−1^. After 30 min at room temperature to allow for acetone volatilisation, the cultures were inoculated with 50–100 *μ*L aliquots of cells grown in M9/MeOH medium to reach an optical density of $\text{O}.\begin{array}{c} \text{D}._{600\;\text{nm}}\sim 0.01 \end{array}$. The cultures were incubated at 28°C under orbital shaking (220 rpm). The control samples were prepared and incubated as described above in the absence of bacteria. After 20 days, the samples were freeze-thawed, and PHE was extracted with 5 mL of hexane by vortexing the mixtures for 5 min. The water and organic phases were separated by centrifugation at 18,000 ×g for 30 min at 4°C. The supernatant organic phase was diluted in hexane, and the PHE concentration was determined spectrophotometrically by measuring the absorbance at 250 nm. A calibration curve was constructed with known PHE concentrations. The abiotic samples were used to calculate the percentage of PHE degradation.

### 2.8. Evaluation of Plant Growth-Promoting Activities

The production of indole acetic acid (IAA) was quantified using the Salkowski colorimetric assay. Briefly, the strains were inoculated in 5 mL of Nutrient Broth (Oxoid) with and without L-tryptophan (2 mg L^−1^, Sigma-Aldrich, Milan, Italy), incubated at 28°C for 7 days, and centrifuged at 6,500 rpm for 15 min. One millilitre of Salkowski's reagent (2 mL of 0.5 M FeCl_3_ with 49 mL of 35% [v/v] HClO_4_) was added to the supernatant (1 mL) and incubated for 30–60 min at room temperature. The IAA concentration was determined by spectroscopic absorbance measurements at 530 nm according to the standard curve.

The Chrome Azurol S (CAS) agar assay was used to evaluate the ability to produce siderophores as described by Silva-Stenico et al. [39]. After 7 days of incubation at 28°C, an orange or yellow halo around the colony indicates the production of siderophores by the microorganisms.

The ability to solubilise phosphates was assayed by inoculating the strains on Pikovskaya's agar (0.5 g L^−1^ of yeast extract, 10 g L^−1^ of dextrose, 0.8 g L^−1^ of Ca_3_(HPO_4_), 0.5 g L^−1^ of (NH_4_)_2_SO_4_, 0.2 g L^−1^ of KCl, 0.1 g L^−1^ of MgSO_4_, 0.0001 g L^−1^ of MnSO_4_, 0.0001 g L^−1^ of FeSO_4_, and 15 g L^−1^ of agar bacteriological) [40]. After incubating the plates at 28°C for 7 days, the capacity of phosphate solubilisation was indicated by clear zones around the colonies.

Ammonia production was tested by inoculating the strain in 5 mL of tryptone water (2%) and incubating it at 28°C for 7 days. The presence of ammonia was detected by the development of a yellow-orange colour after adding a few drops of Nessler's reagent (Sigma-Aldrich) to the broth culture.

All the tests were performed in triplicate.

### 2.9. Plant Germination Test

The surface of the tomato (*Lycopersicon esculentum* L. var.* Jordan*) seeds was sterilised with ethanol (70%) for 10 min and 2.5% (v/v) sodium hypochlorite with 0.05% Tween-20 for 10 min, followed by three or more rinses in sterile H_2_O. The seeds were planted in sterile magenta boxes containing sterile sand irrigated with M9 medium that was supplemented with soil extract (10%) with or without PHE (200 mg L^−1^). The bacterial strain VP2 was inoculated in magenta boxes to evaluate its ability to improve the seeds' germination under stress conditions. The strain was grown at 28°C for 48 h in M9/MeOH (10 mL). Then, the culture was inoculated (1%) in splashboard flasks (200 mL) containing M9/MeOH and incubated under shaking for 48 h in aerobic conditions. The cells were then harvested by centrifugation, washed, and suspended in quarter-strength Ringer's solution (Oxoid) until achieving microbial counts of approximately 5 × 10^8^ CFU mL^−1^ (counting chamber Thoma 0.02 depth, Hawksley UK; 0.5–1 of the McFarland scale). Finally, the VP2 strain was inoculated in magenta boxes to reach a microbial concentration of approximately 1 × 10^6^ cells g^−1^. The control tests were prepared in the absence of PHE and the inoculum.

The plants were cultured in a growth chamber under controlled conditions (constant temperature of 21 ± 1°C, dark/light cycle of 16/8 h d^−1^) for 21 days. Every 7 days, the germinated seeds were counted, which was defined as the germination rate. All the tests were performed in triplicate.

## 3. Results

### 3.1. Isolation and Identification of Potential PAH-Degrading Microorganisms

A total of 14 indigenous microbial isolates that were able to grow in MSSM with CSAE (0.5%) were obtained from soil that was contaminated by different classes of organic compounds (PAHs). Further biotechnological investigations allowed for the selection of the VP2 isolate as the best putative PAH-degrading strain able to grow on MSSM with 1.5% CSAE as the sole carbon source.

The ability of this strain to grow in the highly contaminated habitat was enhanced using a serial enrichment strategy in minimal selective broth media (MSBM) containing increasing concentrations of CSAE. The bacterial strain was able to grow in up to 50% CSAE with and without the addition of nutrients. In fact, after 7 days of incubation at 25°C, VP2 showed an increase of approximately 2 Log CFU mL^−1^ (7.21 ± 0.12 Log CFU mL^−1^) compared to the beginning of the experiment (4.83 ± 0.13 Log CFU mL^−1^) in the presence of nutrients. In the absence of nutrients, the strain showed a smaller increase of up to 5.68 ± 0.10 Log CFU mL^−1^. Moreover, the selected VP2 strain was then identified using a polyphasic approach on the basis of its phenotypic and molecular characterisation. The VP2 colonies were creamy, pink and composed of Gram-negative rod-shaped cells, single or in rosettes. Moreover, this strain was strictly aerobic and tested positive in the catalase and oxidase tests.

**Figure 1:**
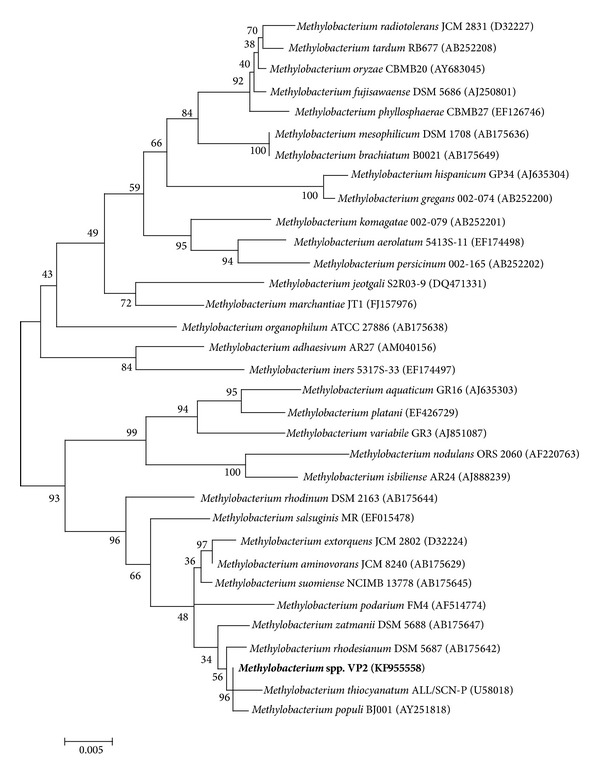
A phylogenetic tree representing the relationship of the 16S rRNA gene sequences of strain VP2 and the type strains of* Methylobacterium* sequences from RDP. Bootstrap values (expressed as percentages of 1,000 replications) are given at the nodes. The sequence accession numbers used for the phylogenetic analysis are shown in parentheses following the species name. The* scale bar* estimates the number of substitutions per site.

The nearly full-length gene sequence (1,409 bp) of this bacterial strain showed an identity of 99% with different* Methylobacterium *species using Blast software. A phylogenetic tree was constructed that included 32 type strains of* Methylobacterium* species. The results of the neighbor-joining analysis of the 16S rRNA sequences of the 32 type strains and VP2 are shown in the dendrogram depicted in Figure 1. The closest relatives of strain VP2 were* Methylobacterium populi, Methylobacterium thiocyanatum, *and* Methylobacterium rhodesianum*.

Because* Methylobacterium* species are closely related and it is difficult to identify new isolates at the species level, biochemical characterisation was performed to ascertain the identity of strain VP2. VP2 was able to use fructose, methane, methylamine, and ethanol as its sole carbon sources, but it was unable to metabolise cyanate, thiocyanate, glucose, and citrate as shown by* M. populi* BJ001^T^ (Table 1). In contrast,* M. thiocyanatum* was able to use cyanate, thiocyanate, glucose, and citrate, and* M. rhodesianum* was unable to use methane. The metabolic profile of VP2 was the same as that shown by* M. populi* BJ001^T^ and differed from the* M. thiocyanatum* and* M. rhodesianum* profiles. Based on these results, the strain VP2 appears to be more similar to* Methylobacterium populi*.

### 3.2. Biofilm Formation on Hydrophobic Surfaces

**Figure 2:**
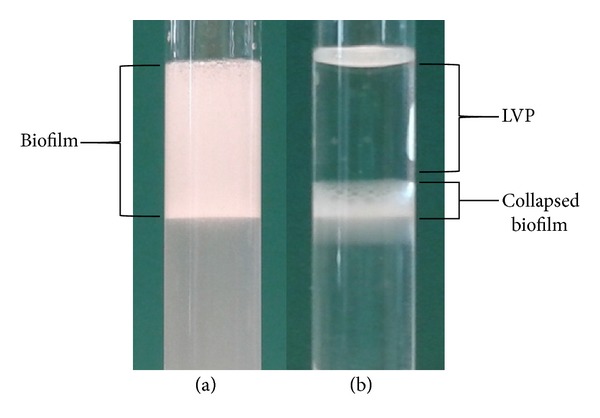
Analysis of the* Methylobacterium populi *VP2 biofilm that formed on LVP/PHE. The sample was collected after 96 h of growth in M9/MeOH liquid broth supplemented with LVP-dissolved phenanthrene. (a): the top phase represents LVP-phenanthrene embedded in a large amount of extracellular polymeric matrix harvested at the end of the growth. (b): the top phase represents LVP-phenanthrene released after freeze-thawing and separation of the phases by centrifugation; the extracellular matrix (collapsed biofilm) forms a disk between the organic phase (top) and water phase (bottom).

To assess the ability of* M. populi* VP2 to grow in a sessile form on hydrophobic surfaces, biofilm growth was performed in an M9/MeOH reach medium broth in the presence of LVP as the hydrophobic phase. To investigate the influence of PAHs on biofilm formation, PHE was dissolved in LVP.* M. populi* VP2 was able to form biofilms on the hydrophobic phase in the absence of PHE. As shown in Figure 2, the cultures grown in the presence of LVP/PHE (Figure 2(a)) or LVP (data not shown) induced a stable emulsification of the oil phase that was completely covered with the extracellular matrix and cells. Freeze-thawing of the emulsified cultures led to the breaking of the biofilm and the releasing of the oil phase. A disk of gelatinous (not homogeneous) material between the aqueous and oil phase was observed (Figure 2(b)). This material could be constructed with the extracellular matrix that was produced from* M. populi* VP2 during its growth in the presence of the hydrophobic phase.

### 3.3. Cell Surface Hydrophobicity

The propensity to grow as a biofilm on hydrophobic surfaces has been often related to the hydrophobicity of the bacterial cell surface, which is believed to be predictive of biofilm formation. However, the CSH test highlighted the fact that* M. populi* VP2 did not show a propensity to adhere to LVP used as a hydrophobic phase. The CSH index value determined for the bacteria grown in the M9/MeOH medium was 1.7%, which was very similar to the value calculated in the presence of LVP (CSH = 2%), suggesting that the presence of LVP alone was unable to change the hydrophobic properties of the cell surface of* M. populi *VP2. It is worth noting that a significant increase of hydrophobicity was observed in the presence of LVP-PHE (CSH = 12%). This finding suggests that the cell surface is modified in the presence of hydrocarbons such as PHE. This feature could be related to the uptake of this hydrophobic substrate during cell growth.

Furthermore, the emulsification test of LVP that was carried out with the culture supernatants allowed for the formation of coalescent drops, and a homogeneous oil phase was separated from the water phase in a few minutes (*E*_24_ = 0%). A similar behaviour was observed when fresh medium broths were tested, thus supporting the inability of the strain to produce bioemulsifiers in all the growth conditions tested.

### 3.4. Phenanthrene Degradation

PHE degradation was investigated in liquid cultures supplemented with PHE microcrystals. The amount of the aromatic hydrocarbon was measured using a spectrophotometer and showed that* M. populi* VP2 degraded approximately 27% of the PHE in 20 days.

### 3.5. Characterisation of Plant Growth Promotion Activities

The selected strain,* M. populi* VP2, was also evaluated for its potential plant growth promotion activities.

The colorimetric assay showed the ability of this strain to produce IAA. In fact, after 7 days of incubation, the strain accumulated the phytohormone in amounts ranging from 1.38 ± 0.07 to 5.27 ± 0.05 mg L^−1^ in the absence and in the presence of L-tryptophan, respectively (Table 2).

Moreover, VP2 was able to solubilise phosphate and to produce siderophores, although in low quantities. In particular, the CAS agar assay showed the ability of the strain to produce siderophores: after incubation, a little yellow halo (1.4 ± 0.03 mm) developed around the colony that was grown under iron-limiting conditions. In addition, the* M. populi* VP2 developed a clear halo with a diameter of 2.8 ± 0.06 mm on a Pikovskaya's agar plate (Table 2), showing a weak ability to solubilise phosphate.

The strain VP2 was not able to produce ammonia because no yellow-orange colour appeared in the broth culture after the addition of Nessler's reagent (Table 2).

### 3.6. Plant Test

To determine whether the methylotrophic bacterial strain could stimulate seed germination, the strain VP2 was inoculated in tomato seeds with and without PHE.* M. populi* VP2 significantly increased the growth and development of the seedlings both in the presence and in the absence of PHE compared to the non-inoculated plants (Table 3). In fact, after 14 days of inoculation, the seeds showed a germination rate of 88.89 ± 11.11%, while the non-inoculated controls showed a germination rate of 14.81 ± 6.41% after 7 days; the control rate remained constant throughout the experiment (Table 3). Moreover, in the presence of PHE, VP2 alleviated PHE phytotoxicity in the tomato seeds. While no seeds germinated in the presence of PHE after 7 days in the magenta box that was inoculated with* M. populi *VP2 or in the non-inoculated control that was treated with PHE, after 14 days, the germination rate in the magenta box was approximately 18% and increased up to 77.78 ± 11.11% by the end of the experiment (after 21 days). This value can be compared to that achieved in the seeds inoculated with* M. populi* VP2 and in the seeds that were not treated with PHE (Table 3). No control seeds that were exposed to PHE stress were able to germinate.

## 4. Discussion

The use of microorganisms is the least expensive and most ecofriendly method used to remediate highly polluted soil. In this study, indigenous microbial strains able to use different classes of polluted organic compounds as their sole carbon source were isolated, characterised, and selected for their potential use in the bioremediation of highly contaminated sites.

Among all the isolates, the strain VP2 that was grown in MSSM with 1.5% CSAE was also able to grow in the presence of up to 50% CSAE. To describe this strain at the species level, it was characterised at the molecular and phenotypic levels by a polyphasic approach. Because this strain was closely related to different* Methylobacterium* species based on the nucleotide sequences of the 16S rRNA gene, a biochemical test was performed as described by Van Aken et al. [41]. When comparing the results of the metabolic profile of the VP2 strain with literature data [41, 42], the* Methylobacterium* spp. VP2 strain appears to be more similar to the* M. populi* species because it was able to use methane [41].

*M. populi* VP2 was selected as the best putative PAH-degrading strain. There are many studies that describe the capacity of several bacteria to mineralise or to degrade the hydrocarbons. Bodour et al. [43] and Andreoni et al. [17] isolated and identified a phenanthrene-degrading culture that contained representative strains belonging to* Methylobacterium *sp. Recently, the biodegradative potential of methylotrophic bacteria toward different polluted compounds, such as explosive, methyl tert-butyl ether (MTBE), and PAHs, has been reported [44–46]. An interesting finding from the current study is that the bacterial species* M. populi *had not previously been reported to be PAH-degrading.* M. populi* VP2 was also able to grow with PHE as its sole carbon and energy source, with a decrease of 27% of the PHE in 20 days. The bacterial strategy utilised to grow on hydrophobic compounds is often related to their ability to form biofilms and produce bioemulsifiers to increase the water solubility of the hydrophobic compounds [47].* M. populi *VP2 was unable to produce bioemulsifiers when grown in the M9/MeOH medium in the presence of LVP or LVP-PHE, thus indicating that emulsifying PHE is not the mechanism for the efficient uptake/degradation of PHE in this strain. However,* M. populi* VP2 that was grown in the M9/MeOH medium in the presence of LVP and LVP-PHE exhibited the ability to form biofilms on the hydrophobic phase that was not related to the LVP-PHE, the emulsifying oil phase, during growth. Similar behaviour was also observed for a* Novosphingobium sp.* strain [37] that was able to emulsify diesel oil by homogeneously coating oil drops with biofilms. To the best of our knowledge, the biofilm formation of* Methylobacterium* on liquid organic phases has not yet been reported even though* Methylobacterium* strains are known to be able to grow in biofilms on living and non-living surfaces [48–52]. Because a crucial event in biofilm formation is the adhesion of the bacteria to a surface, and the hydrophobicity of the microbial surface plays a critical role in the adherence of the bacteria [47], we investigated the hydrophobic features of* M. populi *VP2 cell surface. The assays performed using LVP as a hydrophobic phase highlighted the fact that* M. populi* VP2 cell surface was essentially hydrophilic with CSH index values <20%. However, the CSH values increased from 1.7–2% in the absence of PHE up to 12% when PHE was dissolved in LVP. This finding suggests that the cell surface can undergo changes that are most likely related to the uptake of PHE into the cells. Therefore, the ability of* M. populi* VP2 to form biofilms on hydrophobic surfaces is not related to CSH, but it might be the only opportunity for this strain to interact with hydrophobic substrates because it is unable to produce emulsifiers.

However, the correlation between CSH and biofilm formation has been found to be dependent on the specific strain involved. Bacterial cell hydrophobicity seems to have little or no influence on biofilm formation in* Methylobacterium* sp. and* Mycobacterium mucogenicum* that were isolated from drinking water [50] or on* Staphylococcus epidermidis* [53] and* Candida* strains [54]; however, a positive correlation was observed in 40 clinical* Stenotrophomonas maltophilia* strains tested [55] as well as in corynebacteria that was isolated as part of the natural flora of human skin [56].

*Methylobacterium *sp. has also been shown to interact symbiotically with different plant species of agronomic importance [57] through biofilm formation.* M. extorquens* was endowed with extensive plant-independent biofilm formation, whereas the biofilm formation of* M. mesophilicum* is induced by the plant root [51].

*M. populi* VP2 showed multiple plant growth-promoting traits. In fact, the use of microorganisms with these characteristics is becoming increasingly attractive because they can be used in combination with plants to remediate contaminated environments. Several studies have been conducted on the bioremediation of organic contaminants by PGPR. Huang et al. [23] reported that the removal of PAHs by phytoremediation with the use of PGPR was more rapid and complete than without PGPR because they likely played an important role in increasing plant tolerance to PAHs and promoting plant growth under stress. In fact, soil microorganisms can affect plant growth through the production of hormones, siderophores, and ammonia in addition to improving the plants' mineral supply of phosphorous.

Different studies have reported plant growth-promoting abilities in aerobic methylotrophic bacteria. They are considered to be phytosymbionts and can participate in plant development via the biosynthesis of phytohormones, nitrogen fixation, or by the suppression of ethylene biosynthesis in plants [58], although these activities have never been reported in* M. populi* species. In fact, a clear variability among* Methylobacterium* strains in their capacity to produce the phytohormone IAA has been demonstrated by Omer et al. [59] who reported that only 3 of 16 methylotrophic isolates tested produced IAA under the chosen conditions. The strain* M. populi* VP2 that was obtained in this study presented several desirable features for PGPR. The best of these beneficial features can be observed by the synthesis of IAA. This hormone was used because it is the most common and most efficient among auxins. According to Omer and coworkers [59], the production of IAA increased in the presence of L-tryptophan, resulting in a value almost four times greater. In fact, this amino acid is a precursor of IAA and occurs in plant exudates, stimulating the synthesis of auxins in aerobic methylotrophic bacteria [60]. However, several tryptophan-independent pathways have been described that would explain the production of IAA by* M. populi* VP2 in the absence of this precursor [61]. In addition, the strain VP2 demonstrated other plant growth-promoting activities: it was able to solubilise phosphate and to produce siderophores. The ability of microorganisms to convert insoluble phosphorus to a soluble form is a promising attribute for the selection of bacteria capable of increasing available phosphorus in the rhizosphere [61]. The observed ability of strain* M. populi* VP2 to solubilise calcium phosphate should be related to a process of acidification due to the production of organic acids and/or the secretion of H^+^ or chelating agents, as reported by Anastasio et al. [40]. Jayashree et al. [62] described the ability of 13 pink-pigmented facultative methylotrophic strains belonging to the genus* Methylobacterium* to solubilise phosphate; they demonstrated a phosphate solubilisation index ranging from 1.1 cm to 2.7 cm. Moreover, they observed a significant decline in the pH level coupled with an increase in acidity, indicating that medium acidification is responsible for P-solubilisation.

Another plant growth-promoting activity of* M. populi *VP2 was the production of siderophores, which can influence plant growth by binding Fe^3+^ and making the iron less available for other microorganisms in the rhizosphere, such as plant pathogens [63, 64]. Members of the genus* Methylobacterium*, which are frequently isolated as endophytes from citrus plants, are also able to produce siderophores [65]. Simionato et al. [66] reported and characterised high production of siderophores by* M. mesophilicum* (AR5.1/5 and AR5.1/6 strains) and* M. extorquens* (AR1.6/2 strain). Gholizadeh and Kohnehrouz [67] investigated the presence of Fe-efficient* Methylobacterium *symbiosis in* Celosia cristata*.

In addition, the bacterial strain VP2 was found to have significant and consistent stimulatory effects on tomato seed germination both in the presence and in the absence of PHE contamination. Madhaiyan and coworkers [68] reported the ability of pink-pigmented facultative methylotrophic bacteria (PPFMs) belonging to the genus* Methylobacterium* to promote seed germination and plant growth as well as to induce systematic resistance in rice. Moreover, the phytotoxicity of PAHs, such as PHE, is well recognised in addition to their inhibitory effects on seed germination and the growth of seedlings of different plants [69–72]. In fact, PAH stress can exert adverse effects on growth and the photosynthetic process in addition to causing morphology deformation and changes in enzyme activities [73–76]. In particular, Wei and coworkers [72] reported that germination energy and the germination rate decreased with increasing PHE concentrations in wheat; in particular, the germination rate was 62.5% with 0.05 mg mL^−1^ PHE and was significantly reduced by 68% at high PHE concentrations (0.2 mg mL^−1^). In our study, the germination rate of the tomato seeds was completely inhibited by a PHE concentration of 0.2 mg mL^−1^, but this effect was alleviated by* M. populi *VP2 inoculation, which led to a germination rate of 77.8%.

## 5. Conclusions

The effectiveness of the selective ecological strategy employed in this study allowed for the isolation of indigenous strains that are naturally present in highly contaminated soils. The strain* M. populi *VP2 demonstrated multiple plant growth promotion activities because it was able to produce IAA and siderophores and solubilise phosphate; interestingly, VP2 was able to produce biofilms in the presence of phenanthrene and alleviated PHE stress in tomato seeds. To our knowledge, this is first report describing the simultaneous occurrence of multiple plant growth-promoting activities and the potential biodegradation of xenobiotic organic compounds of industrial origin in* M. populi* VP2. Therefore, this selected strain could be a good candidate to employ in bioremediation strategies to remediate contaminated agricultural soil and may be useful in microbe-assisted phytoremediation.

## Figures and Tables

**Table 1:** Carbon source utilisation in strain VP2, *M.  populi* BJ001^T^ [41], *M.  thiocyanatum* [42], and *M.  rhodesianum *[41].

Carbon source	Strain VP2	*M. populi* BJ001^T^	*M. thiocyanatum *	*M. rhodesianum *
Fructose	+	+	ND	+
Methane	+	+	ND	−
Methylamine	+	+	+	+
Ethanol	+	+	ND	+
Cyanate	−	−	+	ND
Thiocyanate	−	−	+	ND
Glucose	−	−	+	−
Citrate	−	−	+	−

+: growth; −: no growth; ND: not determined.

**Table 2:** Plant growth-promoting activities of *Methylobacterium populi* VP2.

Microbial strain	IAA^a^ production (NB) (mg L^−1^ ± SD)	IAA^b^ production (NB + TRP) (mg L^−1^ ± SD)	Siderophores production	Phosphate solubilization	Ammonia production
*M. populi* VP2	1.38 ± 0.07	5.27 ± 0.05	1.4 ± 0.03 mm	2.8 ± 0.06 mm	—

^a^IAA production in Nutrient Broth without L-tryptophan.

^b^IAA production in Nutrient Broth supplemented with L-tryptophan.

**Table 3:** The effect of phenanthrene (PHE) and *M.  populi *VP2 on the germination rate (%) of tomato seeds^a^.

Time (days)	Control^b^	*M. populi* VP2^c^	PHE^d^	PHE + *M. populi* VP2^e^
7 d	14.81 ± 6.41^A^	37.03 ± 6.41^A^	0 ± 0^C^	0 ± 0^C^
14 d	14.81 ± 6.41^A^	88.89 ± 11.11^B^	0 ± 0^C^	18.52 ± 6.41^A^
21 d	14.81 ± 6.41^A^	92.59 ± 6.41^B^	0 ± 0^C^	77.78 ± 11.11^B^

^a^The values represent the means ± SD of three replicates of independent experiments. Different letters after the values indicate significant differences (*P* ≤ 0.01; *t* test).

^b^Seeds irrigated with M9 medium supplemented with soil extract (10%).

^c^Seeds irrigated with M9 medium supplemented with soil extract (10%) and inoculated with *M.  populi* VP2 (10^6^ cells g^−1^).

^d^Seeds irrigated with M9 medium supplemented with soil extract (10%) and phenanthrene (200 mg L^−1^).

^e^Seeds irrigated with M9 medium supplemented with soil extract (10%) and phenanthrene (200 mg L^−1^) and inoculated with *M.  populi* VP2 (10^6^ cells g^−1^).
